# Antiangiogenic Strategies in Epithelial Ovarian Cancer: Mechanism, Resistance, and Combination Therapy

**DOI:** 10.1155/2022/4880355

**Published:** 2022-04-12

**Authors:** Chengwen Jin, Mingyuan Yuan, Hualei Bu, Chengjuan Jin

**Affiliations:** ^1^Department of Central Laboratory, Shanghai Chest Hospital, Shanghai Jiao Tong University, 241 Huaihai West Road, Shanghai, China 200030; ^2^Department of Obstetrics and Gynecology, Jiaozhou Central Hospital of Qingdao, 29 Xuzhou Road of Jiaozhou, Qingdao, China 266300; ^3^Department of Gynecology and Obstetrics, Qilu Hospital, Cheeloo College of Medicine, Shandong University, Jinan, Shandong, China 250012; ^4^Department of Obstetrics and Gynecology, Shanghai General Hospital, School of Medicine, Shanghai Jiao Tong University, 650 Xinsongjiang Road, Shanghai, China 201600

## Abstract

Angiogenesis is one of the hallmarks of cancer and plays a crucial role in carcinogenesis and progression of epithelial ovarian cancer. Antiangiogenic agent is the first approved targeted agent in ovarian cancer. Anti-angiogenic agents mainly include agents target VEGF/VEGFR pathway, such as bevacizumab and agents target receptor tyrosine kinase, and non-VEGF/VEGFR targets of angiogenesis. Antiangiogenic agents demonstrate certain effects in ovarian cancer treatment either as monotherapy or combined with chemotherapy. Unfortunately, antiangiogenic agents, such as bevacizumab, integrated into the ovarian cancer treatment paradigm do not increase cures. Thus, the benefits of anti-angiogenic agents must be carefully weighed against the cost and associated toxicities. Antiangiogenic agents drug resistance and short of predictive biomarkers are main obstacles in ovarian cancer treatment. A combination of poly (ADP-ribose) polymerase inhibitors or immune checkpoint inhibitors might be great strategies to overcome resistance as well as enhance anti-tumor activity of anti-angiogenic drugs. Predictive biomarkers of antiangiogenic agents are in urgent need.

## 1. Background

Ovarian cancer is one of the most lethal gynecological malignancies [1]. In 2021, there will be approximately 21,410 new ovarian cancer cases diagnosed and 13,770 ovarian cancer deaths in the United States [2]. Ovarian cancer contains a heterogenous group of malignancies that vary in etiology, molecular biology, and numerous other characteristics. 90% of ovarian cancers are epithelial, and the most common subtype of epithelial ovarian cancer is serous carcinoma [3]. Cytoreductive surgery and platinum-based chemotherapy remain the standard therapy for newly diagnosed advanced ovarian cancer patients [4, 5]. Most patients have no evidence of disease after standard treatment, but approximately 70% relapse within the following 3 years [5]. Recurrent ovarian cancer is obviously incurable, and the progression-free survival becomes progressively shorter with the successive treatments given at each subsequent relapse [6]. The most serous carcinoma was diagnosed at advanced stages with stage III (51%) and stage IV (29%). The 5-year overall survival was only 42% for stage III patients and 26% for stage IV patients during 2007 through 2013 [3]. The main reasons for this poor prognosis are the advanced stage at diagnosis, the high rate of disease recurrence, and the eventual emergence of treatment resistance [7].

With the progress in radical surgery and chemotherapy strategies in epithelial ovarian cancer, the 5-year overall survival for advanced ovarian cancer still wanders 40%. It is in urgent need to develop novel treatment options. The molecular-targeted therapies brought hope to precision treatment of ovarian cancer with more specificity and lower toxicity. Antiangiogenic agents played an indispensable role in gynecological cancers. The patients with stage III/IV or recurrent endometrial cancer have a poor prognosis. Thus, active and tolerable novel targeted agents are in an urgent need to improve the prognosis of these patients. The antiangiogenic agents alone or combined with chemotherapy have presented mixed results in treating endometrial cancer patients [8]. The antiangiogenic agent is the first active targeted agent in ovarian cancer. The introduction of the targeted agents has significantly changed the future for the lethal disease. This review summarizes the key clinical trial data on antiangiogenic agents that have led to the current status of treatment of advanced epithelial ovarian cancer.

## 2. Antiangiogenic Agents

Inducing angiogenesis is one of the six hallmarks of cancer acquired during the multistep development of human tumors [9]. Angiogenesis facilitates generation of tumor-associated neovasculature that provides nutrients and oxygen as well as evacuates metabolic wastes and carbon dioxide. In ovarian cancer, angiogenesis induced ascites formation and multiple metastatic spread to promote tumor progression and cause poor prognosis [10]. As such, angiogenesis has been an essential focus for targeted treatment of ovarian cancer.

### 2.1. Agents Target the VEGF/VEGFR Pathway

Vascular endothelial growth factor (VEGF)/VEGF receptor (VEGFR) pathway is one of the most common and important angiogenic pathways in ovarian cancer. VEGF and VEGFR are expressed on ovarian cancer cells, and high expression of VEGF is indicative of unfavorable prognosis [10].

**Figure 1:**
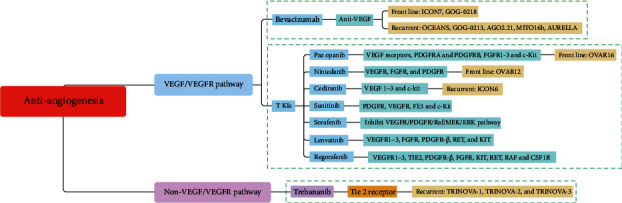
Antiangiogenic drugs used in epithelial ovarian cancer.

Bevacizumab, a humanized anti-VEGF monoclonal antibody, is not only the most widely studied antiangiogenesis agent across distinct tumors, but also the first active targeted agent in ovarian cancer [11]. Many randomized phase III trials adding bevacizumab tinto treatments had been carried out, including bevacizumab in frontline chemotherapy and maintenance (ICON7 [12] and GOG-0218 [13]) , in platinum-sensitive recurrent ovarian cancer (OCEANS Trial [14], GOG-0213 [15], AGO 2.21 [16] and MITO16b [17]), and in platinum-resistant (AURELLA Trial [18]) recurrent epithelial ovarian cancer (Table 1 and Figure 1).

ICON7 and GOG-0218 were two well-known phase III trials first attempt to incorporating bevacizumab in frontline maintenance of ovarian cancer. In ICON7, 7.5 mg per kilogram bevacizumab was used for 12 cycles maintenance which was twice the dose (15 mg per kilogram) bevacizumab for 16 cycles in GOG-0218 [12, 13]. The ICON7 study concluded that bevacizumab improved PFS in ovarian cancer (21.8 months in bevacizumab group VS 20.3 months in standard group, HR: 0.81, 95% CI 0.70-0.94, *P* = 0.004). Moreover, the patients at high risk for progression (FIGO stage IV disease or FIGO stage III disease and >1.0 cm of residual disease after debulking surgery) benefited most from adding bevacizumab to treatment, with PFS of 18.1 months in the bevacizumab group and 14.5 months in the standard group. Moreover, OS for these patients at high risk for progression in bevacizumab group was 36.6 months versus 28.8 months in the standard group. As a result, bevacizumab prolonged 3.6 months of median PFS among patients at high risk for progression [12]. GOG-0218 mainly focused on patients at high risk of progression and uncovered that bevacizumab expanded median PFS about 4 months in ovarian cancer, with 28% reduction in the risk of progression [13]. The concordance in these clinical studies suggests that patients at high risk of progression may be the ideal candidates for frontline bevacizumab.

However, there were concerns on safety of bevacizumab, such as gastrointestinal perforation or fistula, hypertension, venous or arterial thrombosis, and wound disruption [13]. The current dilemma in bevacizumab for high-risk subgroup of advanced ovarian cancer is not cost-effective. A reduction of 46%-67% in the price would be required to make bevacizumab cost-effective in a high-risk subgroup [19]. Moreover, effective biomarkers that predicting survival benefits from bevacizumab was still lacking [20–22], and bevacizumab treatment was associated with decrement in quality of life [23].

Besides primary treatment in ovarian cancer, the efficiency of bevacizumab in recurrent ovarian cancer had been thoroughly explored. The platinum-free interval is not only the most critical prognostic factor for PFS and OS but also determines response to subsequent lines of chemotherapy in patients with recurrent epithelial ovarian cancer. Extending the platinum-free interval with a nonplatinum-based regimen might restore platinum sensitivity to improve survival [24]. AURELIA is the first phase III trial combining bevacizumab with chemotherapy in platinum-resistant ovarian cancer. In AURELIA, the median PFS was 3.4 months in chemotherapy arm versus 6.7 months in bevacizumab-containing arm (HR: 0.48, 95% CI 0.38-0.60, *P* < 0.001). No significant improvement in OS was detected possibly due to crossover to bevacizumab permitted from the chemotherapy subgroup [18]. Based on AURELIA, bevacizumab combined with chemotherapy was considered a standard option in platinum-resistant ovarian cancer.

Four noteworthy randomized phase III trials (OCEANS [14], GOG-0213 [15], AGO 2.21 [16], and MITO16b [17]) concentrated on the addition of bevacizumab to chemotherapy in platinum-sensitive ovarian cancer. In OCEANS, patients recurred >6 months after front-line platinum-based chemotherapy with measurable disease were analyzed and evaluated the efficiency of bevacizumab. The study verified that the addition of bevacizumab to chemotherapy significantly prolonged PFS compared with placebo, with median PFS of 12.4 months and 8.4 months, respectively (HR: 0.484, 95% CI 0.388-0.605, *P* < 0.001) [14]. Since then, incorporation bevacizumab into chemotherapy was regarded as standard regimen in platinum-sensitive ovarian cancer. However, the final median OS in OCEANS was comparable between arms (bevacizumab arm: 33.6 months; placebo arm: 32.9 months; HR: 0.95, *P* = 0.65) [25]. GOG-0213 is an open randomized phase III trial that evaluated bevacizumab and paclitaxel-carboplatin chemotherapy and secondary cytoreduction in recurrent, platinum-sensitive ovarian cancer. Similar to OCEANS, GOG-0213 demonstrated that bevacizumab combination with chemotherapy significantly lengthened median PFS than chemotherapy alone (13.8 months VS 10.4 months, HR: 0.628, 95% CI 0.534-0.739, *P* < 0.001) [15]. Surprisingly, GOG-0213 confirmed that bevacizumab added to standard chemotherapy, followed by maintenance, improved the median OS in platinum-sensitive recurrent ovarian cancer (42.2 months VS 37.3 months, adjusted HR: 0.823, 95% CI 0.680-0.996, *P* = 0.0447) which stands in strong contrast to OCEANS. GOG-0213 initiated the regimen of bevacizumab combination chemotherapy in platinum-sensitive recurrent ovarian cancer patients undergoing secondary cytoreductive surgery [15]. Whether this difference on overall survival between OCEANS and GOG-0213 attributes to different chemotherapy backbones? This needs further prospective validation. AGO-OVAR 2.21/ENGOT-ov18 trial is the first phase 3 trial comparing two bevacizumab-containing regimens in recurrent ovarian cancer. AGO 2.21 trial revealed that both median PFS and OS were superior in the carboplatin-pegylated liposomal doxorubicin- (PLD-) bevacizumab group than the carboplatin-gemcitabine-bevacizumab group, with a median PFS of 13.3 VS 11.6 months (HR: 0.81, 95% CI 0.68-0.96, *P* = 0.012) and median OS of 31.9 VS 27.8 months (HR: 0.81, 95% CI 0.67-0.98, *P* = 0.032) irrespective of the previous antiangiogenic therapy. In AGO 2.21 trial, 41% of patients were previously treated with bevacizumab or other antiangiogenic drugs. The AGO 2.21 trial established carboplatin-PLD-bevacizumab as a new standard treatment option for platinum-eligible recurrent ovarian cancer [16]. MITO16b tested the value of continuing bevacizumab beyond progression after first-line treatment with bevacizumab. The results from MITO16b coincided with that of AGO 2.21. MITO16b demonstrated that bevacizumab combined with chemotherapy improved PFS than chemotherapy alone in platinum-sensitive recurrent ovarian cancer patients that already treated with bevacizumab during first-line therapy (median PFS 11.8 months VS 8.8 months, HR: 0.51, 95% CI 0.41-0.61, *P* < 0.0001) [17].

Unfortunately, all these phase III trials did not assess BRCA mutational status because there was no drug available based on this biomarker at the time of planning the studies.

### 2.2. Agents Target Receptor Tyrosine Kinase

Targeted therapy with tyrosine kinase inhibitors (TKIs) have shown a promise in early phase trials, with several advancing to phase III clinical trials in EOC. Unlike bevacizumab, TKIs engage multiple targets, such as VEGFR, PDGFR, FGFR, c-Kit, and Ret. TKIs are generally administered orally offering increased convenience and flexibility. TKIs seem attractive, but multiple targets may be associated with additional toxicity, uncertain bioavailability, and inflexibility in dosing. Pazopanib, nintedanib, cediranib, sorafenib, sunitinib, lenvatinib, and regorafenib were well-known TKIs in ovarian cancer (Tables 2 and 3 and Figure 1).

#### 2.2.1. Pazopanib

Pazopanib is a TKI of VEGF receptors, PDGFRA and PDGFRB, FGFR1-3, and c-Kit. OVAR16 was a phase III trial evaluating pazopanib maintenance therapy in FIGO II-IV ovarian cancer patients during first-line chemotherapy. OVAR16 demonstrated that pazopanib front-line maintenance significantly prolonged 5.6 months of PFS (HR: 0.77, 95% CI: 0.64-0.91; *P* = 0.0021) [26]. Final analysis revealed no difference in overall survival between pazopanib and placebo (HR: 0.96, 95% CI: 0.805-1.145), and the median OS was 59.1 months and 64.0 months in pazopanib and placebo arms, respectively [27]. Grade 3 or 4 AEs associated with pazopanib were hypertension (30.8%), neutropenia (9.9%), liver-related toxicity (9.4%), diarrhea (8.2%), fatigue (2.7%), thrombocytopenia (2.5%), and palmar-plantar erythrodysesthesia (1.9%). Treatment discontinuation due to AEs was higher among patients treated with pazopanib (33.3%) than with placebo (5.6%) [26]. Exploratory analysis demonstrated that the treatment effect of maintenance pazopanib in East Asian patients seemed to differ from that in non-Asian patients. In East Asian patients, pazopanib maintenance had detrimental effects both on median PFS (HR: 1.114, 95% CI: 0.818-1.518, *P* = 0.4928) and median OS (HR: 1.706, 95% CI: 1.010-2.883, *P* = 0.0465) versus placebo [28]. However, none of the potential factors analyzed could satisfactorily explain the different efficacy results of pazopanib in East Asian patients [28].

Several phase II trials explored the role of pazopanib in recurrent ovarian cancer. MITO 11 meant to assess effects of adding pazopanib in platinum-resistant or platinum-refractory advanced ovarian cancer patients. MTIO 11 suggested that pazopanib combination paclitaxel improved PFS than paclitaxel alone in patients with platinum-resistant or platinum-refractory ovarian cancer (median PFS 6.35 months vs 3.49 months, HR: 0.42, 95% CI: 0.25-0.69, *P* = 0.0002) [29]. Another trial evaluated the combination of pazopanib with weekly gemcitabine in persistent or recurrent ovarian cancer. The study revealed platinum-resistant disease derived the most benefit from combination therapy of pazopanib plus weekly gemcitabine (PFS 5.32 VS 2.33 months, one-sided Tarone-Ware *P* < 0.001) [30]. Whether pazopanib combined weekly paclitaxel or weekly gemcitabine failed to improve the overall survival [29, 30]. PAZOFOS was the first trial investigated pazopanib plus fosbretabulin in relapsed ovarian cancer. However, the trial was discontinued due to cardiac toxicity in the experimental arm [31]. In summary, the results from pazopanib combination in recurrent ovarian cancer were discouraged.

#### 2.2.2. Nintedanib

Nintedanib is a TKI that inhibits VEGFR, FGFR, and PDGFR. A randomized phase II trial assessed the effects and safety of nintedanib maintenance in relapsed ovarian cancer followed chemotherapy [32]. Nintedanib was well tolerated and associated with a potential improvement in PFS. The phase III trial-OVAR12 investigated the combination of nintedanib with standard carboplatin and paclitaxel chemotherapy followed by nintedanib maintenance in patients with newly diagnosed advanced ovarian cancer [33]. PFS in nintedanib group was obviously longer than placebo group (17.2 months versus 16.6 months; HR = 0.84, 95% CI: 0.72-0.98, *P* = 0.024). The post hoc analysis illustrated that non-high-risk subgroups (FIGO stage III and postoperative residuals 1 cm or smaller, or FIGO stage II) benefited most from nintedanib maintenance with PFS 27.1 months in nintedanib group versus 20.8 months in placebo group (HR: 0.74, 95% CI: 0.61-0.91), whereas no difference in PFS was detected in patients with high-risk disease (HR: 0.99, 95% CI: 0.80-1.24). The serious adverse events rate was 42% in nintedanib group and 34% in placebo group. Gastrointestinal was the most common adverse event (diarrhea: nintedanib group 21% grade 3 and <1% grade 4 vs placebo 2% grade 3 only). The updated PFS results were consistent with the primary analysis (HR: 0.86, 95% CI: 0.75-0.98, *P* = 0.029) favoring nintedanib. The final results showed that there was no OS difference between treatments regardless of subgroups (HR: 0.99, 95% CI: 0.83-1.17, *P* = 0.86) [34]. An exploratory analysis revealed that early tumor regrowth facilitated impaired survival in non-high-risk subgroups [35].

Another phase II trial firstly explored efficacy and safety of low dose cyclophosphamide combined nintedanib in relapsed ovarian cancer [36]. It demonstrated that nintedanib did not improve outcomes when added to oral cyclophosphamide. No differences in quality of life between oral cyclophosphamide plus nintedanib group versus oral cyclophosphamide plus placebo group.

#### 2.2.3. Cediranib

Cediranib is an oral TKI of VEGFR 1-3 and c-kit. Two phase II studies were conducted of cediranib to evaluate the safety and effects in the recurrent epithelial ovarian, fallopian tube, and peritoneal cancer. They concluded that cediranib 30 mg daily showed a significant activity in recurrent ovarian cancer, tubal cancer, and peritoneal cancer with manageable toxicities [37, 38]. ICON6 was a phase III trial aiming to assess the efficacy and safety of cediranib maintenance in platinum-sensitive recurrent ovarian cancer [39]. Median PFS was 11.0 months in cediranib 20 mg alongside chemotherapy followed cediranib maintenance and 8.7 months in placebo alongside chemotherapy and then placebo maintenance (HR: 0.56, 95% CI: 0.44–0.72, p <0.0001). When restricting the mean survival time over 3 years, cediranib 20 mg alongside chemotherapy followed by cediranib maintenance arm obtained a 2.9-month improvement compared with placebo alongside chemotherapy and then placebo maintenance arm.(*P* = 0.005). Cediranib was the first oral TKI that improves both PFS and OS in platinum-sensitive recurrent ovarian cancer. During the chemotherapy phase, 32% of patients in the two cediranib arms discontinued cediranib because of the toxic effects compared with 10% in the placebo arm. In the maintenance phase, 10% of patients discontinued cediranib due to toxic effects compared with 2% in placebo arm. The common adverse events during chemotherapy with cediranib were diarrhea, neutropenia, hypertension, and voice changes and during maintenance were diarrhea, hypothyroidism, and voice changes. After treatment of cediranib commenced for 1 year, no quality of life detriment was found [40]. The patients treated with cediranib acquired both maintenance of quality of life and prolonged disease control. Therefore, cediranib played a valuable role in platinum-sensitive recurrent ovarian cancer.

#### 2.2.4. Sunitinib

Sunitinib (SU11248) is an orally administered TKI targeting PDGFR, VEGFR, Flt3, and c-Kit. There were four phase II trials and no phase III trial on sunitinib treatment in ovarian cancer. AGO 2.11 was a phase II trial that evaluated the safety and effectiveness of sunitinib in recurrent platinum-resistant ovarian cancer [41]. The median PFS was 2.9 months in the continuous treatment arm versus 4.8 months in the noncontinuous treatment arm (HR: 1.29, 95% CI: 0.79-2.1, *P* = 0.3048). The median OS was 13.7 months in the continuous treatment arm versus 13.6 months in the noncontinuous treatment arm with no significant difference. The noncontinuous treatment schedule of sunitinib exerted moderate activity in relapsed platinum-resistant ovarian cancer. Hypertension and fatigue were the common adverse events. In platinum-sensitive recurrent ovarian cancer, 50 mg intermittent single-agent sunitinib demonstrated a modest activity with a median PFS of 4.1 months [42], whereas in the recurrent and refractory ovarian, fallopian tube, and peritoneal carcinoma, sunitinib achieved a modest response rate of 8.3% with a median PFS estimated only of 9.9 weeks [43]. GOG-254 evaluated sunitinib in persistent or recurrent clear cell ovarian carcinoma with median PFS and OS of 2.7 months and 12.8 months, respectively. Sunitinib exhibited a minimal activity in the second- and third-line treatment of persistent or recurrent clear cell ovarian carcinoma [44].

#### 2.2.5. Sorafenib

Sorafenib is an oral TKI that inhibits VEGFR/PDGFR/Raf/MEK/ERK pathway. Many phase II trials of sorafenib in ovarian cancer were performed. Sorafenib combined with carboplatin and paclitaxel chemotherapy was not feasible as neoadjuvant regimen in primary ovarian cancer [45]. Sorafenib could not be recommended as a front-line maintenance in patients with ovarian cancer at complete remission [46]. Furthermore, the addition of sorafenib to standard paclitaxel/carboplatin did not improve efficacy compared with standard paclitaxel/carboplatin in the first-line treatment of advanced epithelial ovarian cancer (PFS: 15.4 versus 16.3 months; 2-year survival 76% versus 81%) [47]. Sorafenib failed to achieve a sufficient objective response or sustained disease stabilization as third-line treatment for ovarian cancer [48]. Several trials explored sorafenib in recurrent ovarian cancer. Sorafenib in combination with gemcitabine in recurrent epithelial ovarian cancer achieved a median PFS of 5.4 months and OS of 13 months, but the combination did not meet its primary efficacy end point [49]. Another trial demonstrated that sorafenib had a modest antitumor activity and substantial toxicity in recurrent ovarian cancer [50]. In recurrent platinum-sensitive epithelial ovarian, peritoneal, or fallopian tube cancer, compared with sorafenib alone, sorafenib in combination with carboplatin and paclitaxel improved a response rate (RR) and PFS (RR: 15% VS 61%, *P* = 0.014; PFS 5.6 months VS 16.8 months, *P* = 0.012) [51]. In platinum-resistant ovarian cancer, sorafenib in combination with weekly topotecan resulted in a modest clinical efficacy and increased toxicity [52]. TRIAS further assessed sorafenib combined with topotecan followed by sorafenib maintenance in platinum-resistant ovarian cancer [53]. TRIAS suggested that sorafenib improved both PFS and OS of platinum-resistant ovarian cancer. PFS was significantly improved in the sorafenib group compared with the placebo group (6.7 months VS 4.4 months, HR: 0.6, 95% CI: 0.43-0.83, *P* = 0.0018). Sorafenib obviously prolonged OS compared with placebo (17.1 months VS 10.1 months, HR: 0.65, 95% CI: 0.45-0.93, *P* = 0.017). The promising results from TRIAS supported the essential role of antiangiogenesis as the treatment backbone in combination with chemotherapy, making this approach attractive for further assessment with other targeted strategies.

#### 2.2.6. Lenvatinib

Lenvatinib is an oral multitargeted TKI of VEGFR1-3, FGFR, PDGFR-*β*, RET, and KIT. It has been approved by the FDA in combination with pembrolizumab for microsatellite stable recurrent endometrial cancer in September 2019 [54]. Until now, only one phase I study of lenvatinib which combined weekly paclitaxel in patients with recurrent endometrial, ovarian, fallopian tube, or primary peritoneal cancer was performed. Weekly paclitaxel with lenvatinib shows an encouraging activity and provides a new active option for patients with recurrent platinum-resistant ovarian cancer with manageable side effects [54]. There are four phase II trials and one phase I trial of lenvatinib combined with pembrolizumab or chemotherapy in ovarian cancer registering or recruiting (NCT03797326, NCT04781088, NCT04519151, NCT02788708, and NCT05114421).

#### 2.2.7. Regorafenib

Regorafenib is a multikinase inhibitor targeting VEGFR1-3, TIE2, PDGFR-*β*, FGFR, KIT, RET, RAF, and CSF1R [55]. One phase II trial (REGOVAR) evaluated regorafenib or tamoxifen for platinum-sensitive recurrent ovarian cancer with rising CA125 and no evidence of clinical or RECIST progression. REGOVAR finally found that regorafenib presented an unfavorable toxicity profile with no superior efficacy in these patients [56]. Four phase II trials of regorafenib in ovarian cancer have been registered (NCT05113368, NCT02736305, NCT02278783, and NCT02307500).

### 2.3. Non-VEGF/VEGFR Targets of Angiogenesis

#### 2.3.1. Trebananib

The Ang-Tie pathway plays an important role in blood vessel formation. Angiopoietin 1 (Ang1) and angiopoietin 2 (Ang2) bind to Tie2 receptor to regulate proangiogenic pathways involved in the later stages of neovascularization. Ang 1 promoted vessel stabilization and maturation by recruitment of pericytes to vascular tubes, and Ang 2 enhanced tumor angiogenesis and tumor growth by acting as a vessel destabilizer. Trebananib (formerly known as AMG386) was an investigational recombinant peptide-Fc fusion protein that inhibits tumor angiogenesis by blocking the interaction between Ang1 and 2 and their receptor Tie2 (Figure 1). In a phase I trial of recurrent platinum-resistant or partially platinum-sensitive ovarian cancer, trebananib combined with pegylated liposomal doxorubicin or topotecan showed an evident antitumor activity and acceptable toxicity profiles ([57]). In another phase I trial of patients with ovarian cancer receiving interval or primary debulking surgery, trebananib plus paclitaxel and carboplatin illustrated an antitumour activity and tolerable toxicity [58]. A phase II study of AMG 386 combined with weekly paclitaxel in recurrent ovarian cancer revealed that AMG386 plus weekly paclitaxel improved PFS with a dose-response effect [59]. Unlike agents targeting the VEGF/VEGFR pathway, trebananib had a distinct toxicity profile with peripheral edema being the most frequent adverse events. However, typical anti-VEGF-associated adverse events, such as hypertension, thrombotic events, and gastrointestinal perforations, were not prominent.

Three phase III trials (TRINOVA-1, TRINOVA-2, and TRINOVA-3) (Table 4) explored roles of trebananib in recurrent ovarian cancer and advanced ovarian cancer. TRINOVA-1 suggested that trebananib added into weekly paclitaxel improved PFS significantly in patients with recurrent ovarian cancer (median PFS 7.2 months VS 5.4 months, HR: 0.66, 95% CI: 0.57-0.77, *P* < 0.0001) [60]. The final survival data demonstrated that trebananib prolonged OS only in patients with ascites (14.5 VS 12.3months, HR: 0.72; 95% CI: 0.55-0.93, *P* = 0.011). Moreover, trebananib significantly improved PFS-2 (12.5 VS 10.9 months, HR: 0.85, 95% CI: 0.74-0.98, *P* = 0.024) [61]. TRINOVA-2 aimed to evaluate efficacy of trebananib plus pegylated liposomal doxorubicin in patients with recurrent partially platinum-sensitive or resistant ovarian cancer. Though trebananib added into pegylated liposomal doxorubicin did not improve PFS, the combination improved ORR (objective response rate) and DOR (duration of response) [62]. TRINOVA-3 illustrated that trebananib combined with carboplatin and paclitaxel did not improve PFS as a first-line treatment for advanced ovarian cancer (HR: 0.93, 95% CI: 0.79-1.09, *P* = 0.36) and was not recommended in this population [63].

## 3. Mechanisms of Resistance and Biomarkers of anti-Angiogenic Agents

Bevacizumab was efficacious in only a subset of patients; however, the duration of activity was relatively short, being on the order of 3-8 months with a single-agent therapy. Considering cost, potential toxicity, and limited clinical benefits from antiangiogenic agents, such as VEGF inhibitor bevacizumab, understanding the mechanism of bevacizumab resistance and identifying of predictive biomarkers are of vital importance.

The mechanism of anti-VEGF resistance was comprehensive, including pharmacodynamic tolerance, tachyphylaxis, alteration of the neovascular architecture, redundant angiogenic factors, and induction of hypoxia [64]. Drug tolerance was caused by the increased expression of VEGF and VEGF receptors, changes in signal transduction, or a shift of the stimulus for tumor growth toward other growth factors. Tachyphylaxis referred to an acute decrease in the response to a drug after its administration. Anti-VEGF drug increased intratumoral hypoxia and upregulated HIF-1*α* to induce resistance to bevacizumab [65, 66]. Long-term antiangiogenic therapy significantly alters the expression of angiogenic factors to lead to extensive morphological changes in the vessels. Then, remodeled neovascular architecture resulted in resistance to available antiangiogenic agents [67]. Besides VEGF, many other proangiogenic factors could promote angiogenesis. These factors include fibroblast growth factor (FGF), transforming growth factor, tumor necrosis factor, interleukins, platelet-derived growth factor (PDGF), and placenta growth factor. VEGF signaling was closely linked to other pathways, such as PDGF signaling [68, 69] and FGF signaling [70, 71]. Current antiangiogenic therapy mainly targeted endothelial cells, but recent data indicated that targeting pericytes might provide additional benefits. Pericytes of the vasculature of solid tumors expressed PDGF receptors and acted an important role in tumor vessels. Additionally, PDGF signaling exerted a potential role in regulating immune T microenvironment. PDGF/PDGFR pathway could be the promising drug target for therapeutic intervention [72–75]. FGF interacted with various endothelial cell receptors, such as tyrosine kinase receptors, heparan-sulfate proteoglycans, and integrin to promote tumor growth and angiogenesis. FGF cooperated with VEGF and chemokines to modulate the blood vessel growth in tumor. Moreover, FGF/FGFR system contributed to the onset of mechanisms of resistance to chemotherapy, radiotherapy, and target therapy in tumor. Thus, the FGF/FGFR system represented a potential target for antiangiogenic therapies [76–79]. When VEGF pathway was inhibited, other angiogenic factors or pathways compensatory incresed to stimulate angiogenesis, and final caused resistance to anti-VEGF agents. It has been proven that endothelial p130cas confers resistance to antiangiogenesis therapy and targeting vascular p130cas extends survival of anti-VEGF antibody-resistant ovarian tumors. Thus, p130cas could be a target for overcoming adaptive resistance to antiangiogenic therapy [80]. Identification of resistant mechanism of bevacizumab could provide basis for overcoming drug resistance, improving prognosis and prolonging survival in ovarian cancer patients.

The platinum-free interval (PFI) is the most important prognostic factor for PFS and OS in patients with recurrent ovarian cancer. Platinum-resistant cancers are defined as having a PFI of < 6 months. Platinum resistance is a major impediment in managing ovarian cancer patients. Upregulation of ABCB1, amplification of CCNE1, and presence of BRCA reversion mutations could lead to platinum resistance (2011, [81, 82]). Tumor microenvironment, remarkably immune cell infiltration, angiogenesis, and hypoxia, might induce platinum resistance. Various antiangiogenic agents play an indispensable role in treatment of platinum-resistant ovarian cancers. Bevacizumab with weekly paclitaxel, pegylated liposomal doxorubicin, or topotecan treatment in platinum-resistant ovarian cancers was approved by the FDA based on the AURELIA trial [18]. TKIs such as cediranib demonstrated an obvious activity in platinum-resistant ovarian cancers [38]. Furthermore, the Ang1/2 inhibitor, trebananib, combined with paclitaxel chemotherapy showed an improvement in PFI (7.2 vs 5.4 months, *P* < 0.001) in the TRINOVA-1 trial [60].

Most targeted therapies were used in certain circumstances based on the expression of designated biomarkers, whereas there were no biomarkers to select general patients for the usage of antiangiogenic drugs. Bevacizumab exerted its potential antitumor efficacy only in small proportion patients. Thus, the identification of biomarkers for patient selection and monitoring treatment outcomes of antiangiogenic agents was crucial. To date, no predictive biomarker has been identified and validated that would enable a more personalized and accurate use of bevacizumab. Potential predictive angiogenic markers, such as immunohistochemistry of CD31, TSP-1, VEGF, p53, and ELISA, of circulating levels of VEGF were prospectively examined in the GOG phase II trial of bevacizumab in recurrent and persistent ovarian or peritoneal cancer, but none had been validated [83]. ICON7 evaluated the combined values of circulating Ang1 and Tie2 (Tunica internal endothelial cell kinase 2) concentrations in bevacizumab-treated patients and demonstrated that high Ang1/low Tie2 values were associated with significantly improved PFS (median PFS 23.0 vs 16.2 months, *P* = 0.003). Thus, the ovarian cancer patients with raised plasma concentrations of Ang1 and low Tie2 benefited most from bevacizumab, when concurrently treated with carboplatin and paclitaxel values [84]. Besides combined circulating Ang1 and Tie2, ICON7 also developed a signature comprising mesothelin, FLT4, AGP, and CA-125 to identify ovarian cancer patients benefited more from bevacizumab. It suggested that signature-positive patients had prolonged PFS of 5.5 months [85]. GOG 0262/ACRIN 6695 investigated imaging biomarkers in prediction of prognosis in ovarian cancer. It revealed that early CTP biomarker measurement might provide an early prognostic information for PFS in newly diagnosed ovarian cancer [86]. GOG-0218 analyzed 7 prespecified biomarkers (IL6, Ang-2, osteopontin (OPN), stromal cell-derived factor-1 (SDF-1), VEGF-D, IL6 receptor (IL6R), and GP130) to assess the predictive value of each biomarker with respect to PFS and OS. It illustrated that patients with high IL6 levels treated with bevacizumab had longer PFS (14.2 vs 8.7 months) and OS (39.6 vs 33.1 months) compared with placebo. IL6 might be predictive of therapeutic benefit from bevacizumab when combined with carboplatin and paclitaxel. The additional validation studies are required to determine if IL6 can accurately identify epithelial ovarian cancer patients who may benefit from bevacizumab [20]. In AGO2.11, a high level of circulating Ang-2 was associated with a trend towards unfavorable survival in recurrent ovarian cancer patients (*P* = 0.089). Ang-2 could potentially identify patients that benefited from sunitinib treatment [87]. These findings need to be validated in larger trials due to the limitation of sample size in these studies. The identification of predictive biomarkers remains an urgent medical need in treating epithelial ovarian cancer.

## 4. Future Development of anti-Angiogenic Agents

The combination therapy might be a great strategy to overcome antiangiogenic drug resistance as well as enhance its antitumor activity, though combined therapy might lead to additional toxicities and cost. The novel rationale combinations hold a great promise in enhancing the efficacy of antiangiogenic agents and improving the survival of ovarian cancer patients.

### 4.1. Combination with Immune Checkpoint Inhibitors

Immunotherapy has revolutionized the treatment of cancer, enabling durable control of previously incurable and highly aggressive cancers, being one the most robust and promising area of clinical discovery in solid tumors. Immune checkpoint inhibitors (ICIs) demonstrate an outstanding efficacy against various cancers through reactivating dysfunctional or exhausted T cells [88, 89]. The current approved ICIs mainly consist of anti-cytotoxic T lymphocyte-associated protein 4 (anti-CTLA-4) [90], antibodies against programmed cell death 1 (PD-1), and its ligand (PD-L1) [91]. The majority of patients with tumors did not benefit from immune-checkpoint inhibitors and would experience severe adverse events [92]. The accurate mechanism of the unconventional pattern of clinical response to ICIs has not been clarified. The biomarkers predicting responsiveness to ICIs have been widely investigated to guide future precision immunotherapy [93].

The antiangiogenic agents improved treatment outcomes mainly through normalization of the abnormal tumor vasculature. The tumor vascular normalization could increase the infiltration of immune effector cells into tumors and convert the intrinsically immunosuppressive tumor microenvironment (TME) to an immunosupportive one. Immunotherapy depended on the accumulation and activity of immune effector cells within the TME. Thus, immune responses and vascular normalization seemed to be reciprocally regulated [91]. The anti angiogenic therapy could improve immunotherapy outcomes due to the inhibition of various immunosuppressive features of angiogenesis [94]. The combination therapy with immune checkpoint blockade and antiangiogenic strategy demonstrated an improved anticancer efficacy and prolonged survival [95].

Most clinical trials on ICIs in ovarian cancer were in phase I and phase II. Disappointed, ORR for advanced or recurrent ovarian cancer treated by ICIs alone was relatively not high, ranging from 5.9% to 22.2% [96–103]. The phase III study JAVELIN Ovarian 200 revealed that avelumab alone or in combination with chemotherapy versus chemotherapy alone did not improve PFS or OS in platinum-resistant or platinum-refractory ovarian cancer patients [102]. In short, ICIs alone or combined with chemotherapy showed a poor performance in treatment of ovarian cancer.

Studies focused on bevacizumab plus ICIs were in phase I and phase II, with ORR ranging from 15% to 32%[104–106], which was obviously higher than ICIs alone. A phase Ib study demonstrated that ORR of atezolizumab plus bevacizumab in platinum-resistant ovarian cancer was 15% [106]. A phase II study evaluated the clinical activity associated with the combination of nivolumab and bevacizumab in women with recurrent epithelial ovarian cancer. The study revealed that ORR was 40.0% (19.1%-64.0%) in platinum-sensitive and 16.7% (95% CI 3.6%-41.4%) in platinum-resistant participants [105]. The phase II LEAP-005 study evaluated efficacy and safety of lenvatinib, an antiangiogenic multiple receptor tyrosine kinase inhibitor, plus pembrolizumab in patients with heavily pretreated ovarian cancer. The results from LEAP-005 suggested that the combination achieved ORR of 32% and manageable treatment-related adverse events [104]. The antiangiogenic agents plus immune checkpoint inhibitors illustrated an encouraging efficacy and manageable safety. Therefore, the phase III randomized trials of combination therapy are imminent.

An immune checkpoint upregulation is inextricably linked to cancer-induced angiogenesis. Co-applied antiangiogenic drugs with ICIs approved by the FDA have already provided an exciting efficacy for certain malignancies. However, the inefficiencies in tumor penetrance and increased adverse events were obstacles in this ICIs/antiangiogenic combination therapy. The novel agents such as engineered antibodies may help further springboard the already favorable outcomes of ICIs/antiangiogenic strategies in patients [107].

### 4.2. Combination with Poly (ADP-Ribose) Polymerase Inhibitors

The poly (ADP-ribose) polymerase (PARP) inhibitors (PARPi) are a promising class of drugs that exhibit a remarkable antitumor activity against ovarian cancer. PARPi functions its antitumor activity mainly through the mechanism of synthetic lethality. In tumor with homologous recombination deficiency (HRD), the inhibition of PARP by PARPi leads to the accumulation of double-stranded DNA breaks that cannot be accurately repaired, resulting in synthetic lethality [108, 109]. BRCA1 and BRCA2 play crucial roles in DNA double-strand break repair by homologous recombination, and prevalence of BRCA1/2 mutation in patients with newly diagnosed high-grade serous ovarian cancer is 20-25% (2011, [109–112]). HRD is not limited to tumors with BRCA mutations and is present in approximately 50% of high-grade serous ovarian tumors (1).

Olaparib maintenance treatment provided significant PFS and OS benefits in patients with platinum-sensitive, relapsed ovarian cancer and a BRCA1/2 mutation [6, 113]. Moreover, olaparib maintenance therapy brought substantial PFS benefits with a 70% lower risk of disease progression or death in patients with newly diagnosed advanced ovarian cancer and a BRCA1/2 mutation [114]. In addition, niraparib maintenance therapy also induced longer PFS both in patients with platinum-sensitive, recurrent, and newly diagnosed advanced ovarian cancer, regardless of the presence or absence of BRCA mutation or HRD status [115, 116]. However, among patients with primary or recurrent ovarian cancer and BRCA1/2 wildtype, especially HRD (-), survival benefits from olaparib or niraparib were relatively limited.

Preclinical studies suggest that angiogenesis inhibitors combined with PARPi demonstrate supra-additive effects. Hypoxia induced downregulation of homologous recombination repair genes, such as BRCA1 and RAD51, which could enhance PARPi sensitivity [117, 118]. A phase II trial compared cediranib combined olaparib versus olaparib alone in recurrent platinum-sensitive ovarian cancer [119]. Median PFS was 17.7 months for patients treated with cediranib plus olaparib compared with 9.0 months for those treated with olaparib monotherapy (HR: 0.42, 95% CI 0.23-0.76; *P* = 0.005). ORR was 47.8% with a single-agent olaparib compared to 79.6% with cediranib plus olaparib (OR: 4.24, 95% CI: 1.53–12.22, *P* = 0.002). A post hoc exploratory analysis suggested that an increased activity was higher in gBRCAwt/u patients than in gBRCAm patients. The underlying mechanism might be that cediranib could increase tumor hypoxia and inhibit platelet-derived growth factor receptor to downregulate BRCA1/2 and RAD51, thus decreasing a homology-deficient DNA repair to confer olaparib sensitivity [120]. Approximately 70% of patients in the olaparib/cediranib arm experienced a grade 3/4 toxicity. The latest randomized phase II trial compared PFS in weekly paclitaxel vs. cediranib-olaparib in platinum-resistant ovarian cancer. The results demonstrated that cediranib-olaparib oral doublet was active and might offer a nonchemotherapy option in these population, though combination of cediranib-olaparib was not superior to chemotherapy in PFS [121]. A phase II trial NSGO-AVANOVA2/ENGOT-ov24 investigated niraparib plus bevacizumab for platinum-sensitive recurrent ovarian cancer [122]. The median PFS was 11.9 months in niraparib plus bevacizumab compared with 5.5 months in niraparib alone in patients with platinum-sensitive recurrent ovarian cancer (HR: 0.35, 95% CI 0.21-0.57, *P* < 0.0001). Grade 3 or worse adverse events occurred in 65% patients who received niraparib plus bevacizumab and 45% who received single-agent niraparib. These combinations deserve phase III trials, but toxicity might be problematic. A phase III trial PAOLA-1 evaluated the effects of olaparib plus bevacizumab as a first-line maintenance in newly diagnosed, advanced, high-grade ovarian cancer [123]. The median PFS was 22.1 months with olaparib plus bevacizumab group and 16.6 months with placebo plus bevacizumab group (HR: 0.59, 95% CI 0.49-0.72, *P* < 0.001). The subgroup analyses were performed based on BRCA mutation and HRD status. The median PFS was 37.2 vs. 17.7 months for HRD-positive patients, including BRCA mutations (HR: 0.33, 95% CI: 0.25-0.45). In patients with HRD-positive tumors that did not have BRCA mutations, the median PFS was 28.1 vs. 16.6 months (HR: 0.43, 95% CI: 0.28-0.66). The final analysis of PAOLA-1 proved that olaparib plus bevacizumab maintenance improved PFS significantly in HRD-positive patients with a reduction of risk of progression or death of 61% in the higher-risk group and of 85% in the lower-risk group compared with bevacizumab alone [124]. The addition of olaparib to bevacizumab did not increase the known toxic effects associated with bevacizumab. Olaparib combined bevacizumab as first-line maintenance provided substantial PFS benefits in HRD-positive patients, regardless of the BRCA status.

## 5. Conclusions

Angiogenesis is crucial for the outgrowth of cancers. Antiangiogenic agents proved to play an indispensable role in gynecological cancers. Antiangiogenic agents contain three main categories: agents target the VEGF/VEGFR pathway, agents target receptor tyrosine kinase, and non-VEGF/VEGFR targets of angiogenesis. Bevacizumab is the first active targeted agent that targeted the VEGF/VEGFR pathway approved by the FDA in ovarian cancer. The representative TKIs in ovarian cancer mainly include pazopanib, nintedanib, cediranib, sorafenib, sunitinib, lenvatinib, and regorafenib. Trebananib belongs to the agents of non-VEGF/VEGFR targets of angiogenesis.

Almost all phase III trials of bevacizumab showed that bevacizumab could significantly improve the PFS in patients of recurrent ovarian cancer irrespective of sensitivity of platinum. However, bevacizumab failed to improve OS in ovarian cancer patients. Similar to bevacizumab, various TKIs, such as pazopanib, nintedanib, cediranib and sorafenib prolonged PFS of ovarian cancer. Only two phase II trials of TKIs demonstrated significant improvement of OS in ovarian cancer. One was that sorafenib plus topotecan improved OS of recurrent platinum-resistant ovarian cancer by 7 months versus placebo plus topotecan (HR: 0.65, 95% CI: 0.45–0.93). The other was that pazopanib combined fosbretabulin improved OS of recurrent ovarian cancer compared with pazopanib alone (HR: 0.1, 95% CI: 0.01-0.91). More and more patients have access to antiangiogenic agents. The usage of antiangiogenic agents does not increase the cures. Biomarkers to pick out patients who benefit most from antiangiogenic agents are in short. Antiangiogenic agents are associated with significant more toxicity and higher expenses which limit its promotion and clinical application. Thus, identifying optimal biomarkers for patients benefiting most from antiangiogenic agents is urgent and of vital importance.

Immunotherapy has been the most promising area of clinical discovery in solid tumors. ICIs demonstrate excellent antitumor efficacy through reactivating dysfunctional or exhausted T cells. Immunotherapy relied on the accumulation and activity of immune effector cells within the TME. Tumor-associated neovasculature affected the infiltration of immune effector cells and TME. Thus, immune responses and angiogenesis were reciprocally regulated. The FDA has approved antiangiogenic drugs combined ICIs for certain malignancies due to improved antitumor efficacy. Phase III trials of this combination therapy are imminent in ovarian cancer.

PARPi is a prospective class of drugs that exhibit remarkable antitumor activity against ovarian cancer through the mechanism of synthetic lethality. PARPi exerted the most notable efficacy in ovarian cancer patients carrying BRCA1/2 mutations, followed by HRD (+) patients. However, as for patients with primary or recurrent ovarian cancer and BRCA1/2 wildtype, especially HRD (-), survival benefits from PARPi were relatively limited. Antiangiogenic agents could induce hypoxia, and then hypoxia induced downregulation of homologous recombination repair genes, such as BRCA1 and RAD51, which could enhance PARPi sensitivity. Phase III trials confirmed the synergized efficacy of antiangiogenic agents combined PARPi in BRCA1/2 wildtype, especially HRD (-) ovarian cancer patients.

A combination of PARPi or immune checkpoint inhibitors would help overcome antiangiogenic drug resistance and enhance its antitumor activity. The optimal combinations and predictive biomarkers urgently need further exploration.

## Figures and Tables

**Table 1:** Phase III trials of bevacizumab in ovarian cancer.

Study	Setting	*N*	Treatment arm	PFS (median months)	PFS-HR (95% CI)	OS (median months)	OS-HR(95% CI)
GOG-218	Front-line and maintenance	1873	I: chemotherapy with placebo added in cycles 2 through 22	10.3	—	41.1	—
			II: chemotherapy with bevacizumab added in cycles 2 through 6 and placebo added in cycles 7 through 22	11.2	0.908 (0.795-1.040)	40.8	1.06 (0.94-1.20)
			III: chemotherapy with bevacizumab added in cycles 2 through 22	14.1	0.717 (0.625-0.824)	43.4	0.96 (0.85-1.09)
ICON7	Front-line and maintenance	1528	I: paclitaxel + carboplatin	17.4	0.87 (0.77-0.99)	44.6	0.99 (0.85-1.14)
			II: paclitaxel + carboplatin + bevacizumab; bevacizumab maintenance	19.8		45.5	
OCEANS	Platinum-sensitive recurrent	484	I: chemotherapy (gemcitabine and carboplatin)	8.4	0.484 (0.388-0.605)	29.9	0.751 (0.537-1.052)
			II: bevacizumab with chemotherapy	12.4		35.5	
AURELLA	Platinum-resistant recurrent	361	I: single-agent chemotherapy (pegylated liposomal doxorubicin, weekly paclitaxel, and topotecan)	3.4	0.48 (0.38-0.60)	13.3	0.85 (0.66-1.08)
			II: single-agent chemotherapy + bevacizumab	6.7		16.6	
GOG-213	Recurrent, platinum-sensitive	674	I: paclitaxel + carboplatin	10.4	0.628 (0.534-0.739)	37.3	0.829 (0.683-1.005)
			II: paclitaxel + carboplatin + bevacizumab	13.8		42.2	
AGO 2.21	Platinum-sensitive recurrent	682	I: carboplatin + gemcitabine + bevacizumab; bevacizumab maintenance	11.6	0.81 (0.68-0.96)	27.8	0.81 (0.67-0.98)
			II: carboplatin + pegylated liposomal doxorubicin + bevacizumab; bevacizumab maintenance	13.3		31.9	
MITO 16b	Platinum-sensitive recurrent	406	I: carboplatin-based doublet intravenously	8.8	0.51 (0.41-0.65)	27.1	0.99 (0.73-1.39)
			II: carboplatin-based doublet plus bevacizumab	11.8		26.7	

**Table 2:** Characteristics of phase II and III trials of TKIs in ovarian cancer.

Study	Year	Stage	Targeting agent	Setting
AG02.11	2012	Phase II	Sunitinib	Recurrent platinum-resistant
OVAR 16	2014	Phase III	Pazopanib	Front-line and maintenance
MITO 11	2015	Phase II	Pazopanib plus weekly paclitaxel	Platinum-resistant or platinum-refractory
OVAR 12	2016	Phase III	First-line chemotherapy with or without nintedanib	Front-line and maintenance
ICON6	2016	Phase III	Cediranib	Recurrent platinum-sensitive
TRIAS	2018	Phase II	Sorafenib plus topotecan	Recurrent platinum-resistant
PAZOFOS	2020	Phase II	Pazopanib and fosbretabulin	Recurrent
NCT01610206	2020	Phase II	Weekly gemcitabine plus pazopanib	Persistent or recurrent
NCT01610869	2020	Phase II	Cyclophosphamide and nintedanib	Recurrent
REGOVAR	2022	Phase II	Regorafenib or tamoxifen	Recurrent platinum-sensitive
NCT00710762	2011	Phase II	Nintedanib	Recurrent

**Table 3:** Phase II and III trials of TKIs in ovarian cancer.

Study	*N*	Treatment arm	PFS (median months)	PFS-HR (95% CI)	OS (median months)	OS-HR (95% CI)
AGO 2.11	73	Noncontinuous treatment arm	4.8	0.91 (0.62–1.32)	13.6	0.95 (0.55-1.63)
		Continuous treatment arm	2.9		13.7	
OVAR16	940	Placebo	12.3	0.77 (0.64 - 0.91)	64	0.96 (0.805-1.145)
		Pazopanib	17.9		59.1	
MITO 11	74	Paclitaxel	3.49	0.42 (0.25-0.69)	13.7	0.60 (0.32-1.13)
		Paclitaxel and pazopanib	6.35		19.1	
OVAR 12	1503	Standard carboplatin and paclitaxel chemotherapy	16.6	0.84 (0.72-0.98)	62.8	0.99 (0.83-1.17)
		Nintedanib with standard carboplatin and paclitaxel chemotherapy	17.2		62	
ICON6	486	Chemotherapy + placebo; placebo maintenance	8.7		21	
		Chemotherapy + cediranib; placebo maintenance	9.9	—	NS	—
		Chemotherapy + cediranib; cediranib maintenance	11	0.56 (0.44-0.72)	26.3	0.77 (0.55-1.07)
TRIAS	185	Placebo plus topotecan	4.4	0.60 (0.43-0.83)	10.1	0.65 (0.45–0.93)
		Sorafenib plus topotecan	6.7		17.1	
PAZOFOS	21	Pazopanib	3.7	0.30 (0.09-1.03)	8.4	0.1 (0.01-0.91)
		Pazopanib and fosbretabulin	7.6		NR	
NCT01610206	148	Weekly gemcitabine	2.9	0.61 (0.40-0.92), 1.50(0.76-2.94)	15.6	NS
		Weekly gemcitabine plus pazopanib	5.3		14.2	
NCT01610869	117	Oral cyclophosphamide plus placebo	2.6	0.91 (0.62-1.32)	6.4	1.08 (0.72-1.62)
		Oral cyclophosphamide plus nintedanib	2.9		6.8	
REGOVAR	68	Regorafenib	4.6	1.21 (0.78–1.86)	NR	1.32 (0.70–2.47)
		Tamoxifen	5.6			
NCT00710762	83	Nintedanib	16.3%^#^	0.65 (0.41-1.02)	NS	0.84 (0.51-1.39)
		Placebo	5.0%^#^		NS	

^#^PFS rate at 36 weeks; NR: not reached; NS: not stated; ^#^0.61 (0.40-0.92) during the first 6 months, 1.50 (0.76-2.94) thereafter.

**Table 4:** Phase III trials of trebananib in ovarian cancer.

Study	Year	Setting	*N*	Treatment arm	PFS (median months)	PFS-HR (95% CI)	OS (median months)	OS-HR (95% CI)
TRINOVA-3	2019	First-line treatment	1164	Placebo plus carboplatin and paclitaxel	15	0.93 (0.79-1.09)	43.6	0.99 (0.79-1.25)
				Trebananib plus carboplatin and paclitaxel	15.9		46.6	
TRINOVA-1	2014	Recurrent ovarian cancer	919	Weekly paclitaxel plus placebo	5.4	0.66 (0.57-0.77)	18.3	0.95 (0.81-1.11)
				Weekly paclitaxel plus trebananib	7.2		19.3	
TRINOVA-2	2017	Partially platinum sensitive or resistant	223	PLD plus placebo	7.2	0.92 (0.68-1.24)	17	0.94 (0.64-1.39)
				PLD plus trebananib	7.6		19.4	

## Data Availability

All data used to support the findings of this study are included within the article.
